# Laparoscopic liver resection is associated with less significant muscle loss than the conventional open approach

**DOI:** 10.1186/s12957-022-02854-1

**Published:** 2022-12-04

**Authors:** Ruoh-Yun Gau, Hsin-I Tsai, Ming-Chin Yu, Kun-Ming Chan, Wei-Chen Lee, Haw-En Wang, Sheng-Fu Wang, Mei-Ling Cheng, Chien-Chih Chiu, Hsin-Yi Chen, Chao-Wei Lee

**Affiliations:** 1 Division of General Surgery, Department of Surgery, Linkou Chang Gung Memorial Hospital, Guishan, Taoyuan, 33305 Taiwan; 2 Graduate Institute of Clinical Medical Sciences, Chang Gung University, Guishan, Taoyuan, Taiwan; 3 College of Medicine, Chang Gung University, Guishan, Taoyuan, Taiwan; 4 Department of Anesthesiology, Linkou Chang Gung Memorial Hospital, Guishan, Taoyuan, Taiwan; 5 Division of General Surgery, Department of Surgery, New Taipei Municipal Tu-Cheng Hospital (built and operated by Chang Gung Medical Foundation), Tu-Cheng, New Taipei City, Taiwan; 6 Department of Gastroenterology and Hepatology, Linkou Chang Gung Memorial Hospital, Guishan, Taoyuan, Taiwan; 7 Metabolomics Core Laboratory, Healthy Aging Research Center, Chang Gung University, Guishan, Taoyuan, Taiwan; 8 Department of Biomedical Sciences, Chang Gung University, Guishan, Taoyuan, Taiwan; 9 Clinical Metabolomics Core Laboratory, Linkou Chang Gung Memorial Hospital, Guishan, Taoyuan, Taiwan; 10 Department of Nursing, Linkou Chang Gung Memorial Hospital, Guishan, Taoyuan, Taiwan; 11 Department of Cancer Center, Linkou Chang Gung Memorial Hospital, Guishan, Taoyuan, Taiwan

**Keywords:** Laparoscopic, Liver resection, Hepatectomy, Hepatocellular carcinoma, Sarcopenia, Muscle loss

## Abstract

**Background:**

Laparoscopic liver resections (LLR) have been shown a treatment approach comparable to open liver resections (OLR) in hepatocellular carcinoma (HCC). However, the influence of procedural type on body composition has not been investigated. The aim of the current study was to compare the degree of skeletal muscle loss between LLR and OLR for HCC.

**Methods:**

By using propensity score matching (PSM) analysis, 64 pairs of patients were enrolled. The change of psoas muscle index (PMI) after the operation was compared between the matched patients in the LLR and OLR. Risk factors for significant muscle loss (defined as change in *PMI* > mean change minus one standard deviation) were further investigated by multivariate analysis.

**Results:**

Among patients enrolled, there was no significant difference in baseline characteristics between the two groups. The PMI was significantly decreased in the OLR group (*P* = 0.003). There were also more patients in the OLR group who developed significant muscle loss after the operations (*P* = 0.008). Multivariate analysis revealed OLR (*P* = 0.023), type 2 diabetes mellitus, indocyanine green retention rate at 15 min (ICG-15) > 10%, and cancer stage ≧ 3 were independent risk factors for significant muscle loss. In addition, significant muscle loss was associated with early HCC recurrence (*P* = 0.006). Metabolomic analysis demonstrated that the urea cycle may be decreased in patients with significant muscle loss.

**Conclusion:**

LLR for HCC was associated with less significant muscle loss than OLR. Since significant muscle loss was a predictive factor for early tumor recurrence and associated with impaired liver metabolism, LLR may subsequently result in a more favorable outcome.

**Supplementary Information:**

The online version contains supplementary material available at 10.1186/s12957-022-02854-1.

## Background

Liver resection remains one of the major curative treatments for hepatocellular carcinoma (HCC) [1]. With the improvement in surgical technique and instruments, laparoscopic liver resection (LLR) has gradually become an acceptable approach for surgical resection of HCC. The surgical outcome between laparoscopic and conventional open liver resection (OLR) for HCC had been widely investigated. In addition to comparable oncological outcome, the potential benefits of LLR also included shorter postoperative hospital stay, reduced blood loss, and less occurrence of postoperative complications [2–7]. LLR, as a result, has been endorsed by many experts and consensus to be a justified treatment approach for the management of HCC.

Sarcopenia, initially described and noticed in elderly people with a negative impact on health, is a syndrome characterized by physical frailty, malnutrition, and loss in muscle volume and strength [8, 9]. It is recognized nowadays that not only people with aging or disability but also those suffering from diseases, metabolic disorders, or stress events are also at risk of developing sarcopenia [10, 11]. In the meanwhile, the significant role of sarcopenia in various clinical conditions including cancers, critical illness, trauma, and major surgery has also been demonstrated [12–16]. For example, studies have shown that preoperative sarcopenia could independently predict adverse outcomes following major abdominal operations [14, 17–19]. For patients undergoing liver resection, preoperative sarcopenia significantly increased postoperative complications and impaired long-term survival [15, 20, 21]. Moreover, loss of skeletal muscle mass was found associated with all-cause mortality and tumor recurrence in patients with HCC [21]. In addition to baseline body composition, there were other studies reporting the negative impact of dynamic change, i.e., treatment-related accelerated muscle loss, on the surgical outcome of various cancers [22–26].

Although sarcopenia was significantly correlated with an adverse outcome following liver resections, the influence of procedural type on body composition in patients with HCC has not been fully investigated. Compared to the open procedure, it is widely accepted that the smaller incisions inherent to LLR may contribute to less wound pain, earlier ambulation, and shorter hospital stay after the surgery [4, 5, 27, 28]. In addition, the systemic stress response produced by the laparoscopic approach was also less than the conventional open approach [29, 30]. Consequently, we theoretically hypothesized that these intrinsic advantages would reduce the adverse effect of surgery on body composition and further improve treatment outcome. The aim of the current study was thus to compare the degree of skeletal muscle loss between LLR and OLR for HCC. The influence of skeletal muscle loss on serum metabolome and survival outcome was also investigated.

## Materials and methods

### Patients

Under the approval of the Institutional Review Boards (IRB) of Chang Gung Memorial Hospital (CGMH) (Nos. 201600940B0, 201602025B0, and 202100068B0), a total of 696 patients who received liver resection for primary HCC in Linkou CGMH from 2012 to 2019 were reviewed. The exclusion criteria were as follows (1) patients who lacked critical clinical data or image, (2) who received concomitant extrahepatic surgery except cholecystectomy, (3) who underwent intraoperative vascular/biliary reconstruction, (4) who died within 30 days of surgery, and (5) who had history of major operations or trauma within 3 months of liver surgery. Five-hundred and ninety-six patients, of whom 90 received LLR and 506 underwent OLR, remained and were enrolled for further analysis.

**Fig. 1:**
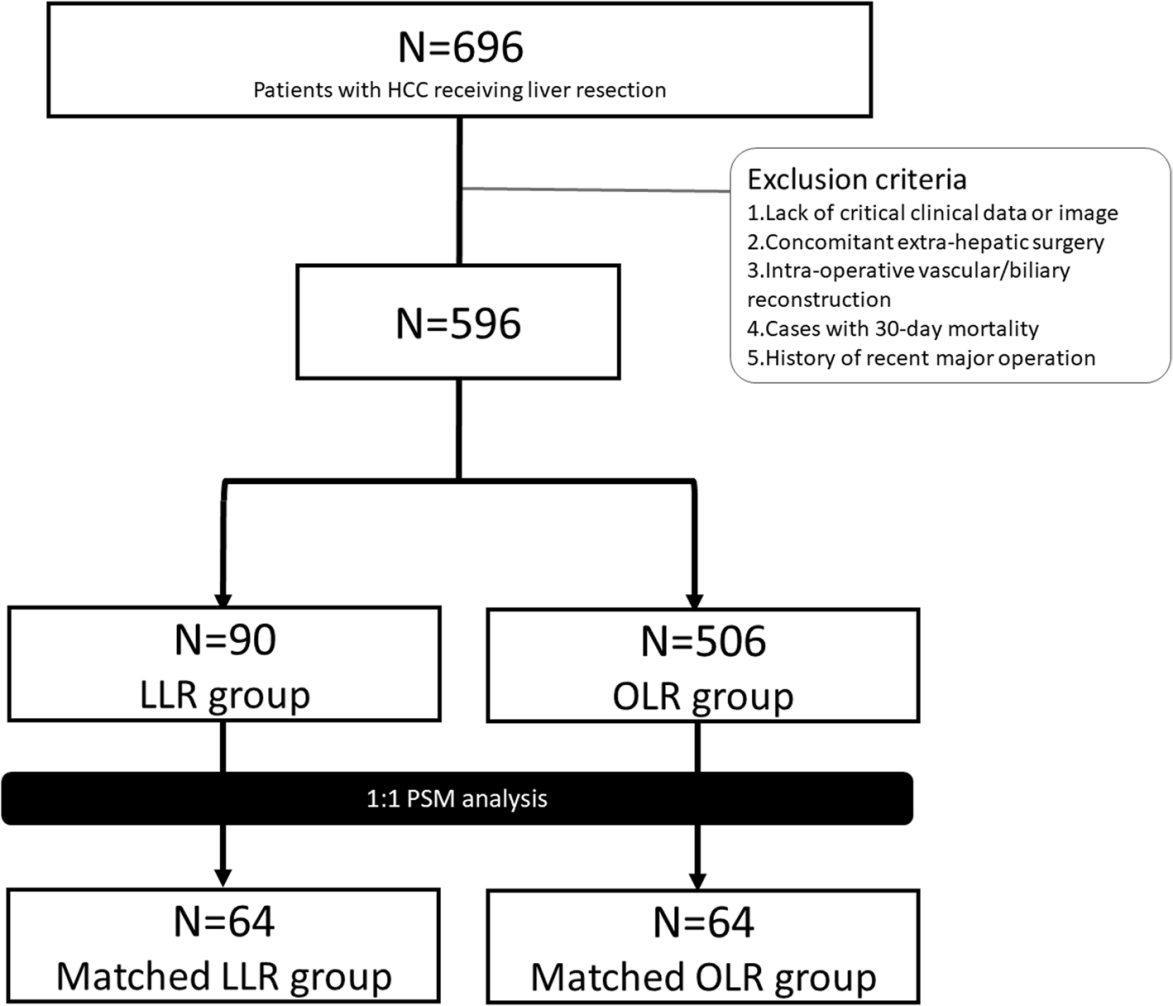
Study flow chart of the current study

Demographic data including age, gender, cigarette smoking, alcohol consumption, hepatitis B virus (HBV) infection, hepatitis C virus (HCV) infection, serum bilirubin, albumin, prothrombin time, international normalized ratio (INR), alanine aminotransferase (ALT), aspartate aminotransferase (AST), gamma-glutamyltransferase (GGT), platelet count, platelet-to-lymphocyte ratio (PLR), prognostic nutritional index (PNI = serum albumin level (in grams per liter) + 0.005 × lymphocyte count (in per mm^3^)) [31, 32], cholesterol, and alpha-fetoprotein (AFP) were recorded from the prospectively established database. The surgical variables, pathological factors, and oncological outcome including tumor size, American Joint Committee on Cancer (AJCC) stage, history of previous abdominal surgery, extent of liver resection, tumor encapsulation, histological grade, vascular invasion, daughter nodules, resection margin, liver cirrhosis, hepatitis activity index (HAI), tumor recurrence, and long-term survival were also recorded. The demographic characteristics of the entire cohort were summarized in Supplementary Table S1. A 1:1 propensity score matching (PSM) analysis between the LLR and OLR groups was then adopted. The variables employed for PSM included tumor size, AJCC cancer stage, previous abdominal surgery, IWATE difficulty score, and extent of liver resection. Sixty-four matched pairs were finally identified and analyzed in the current study (Fig. 1). Tumor was staged by the 7th edition of AJCC TNM staging system for HCC in the current study [33, 34].

### Assessment of muscle

For preoperative staging workup and postoperative follow-up, all patients recruited had undergone computed tomography (CT) scans both before and around 1 month after liver resections. The median interval between liver resections and the first postoperative CT scan was 38 days (interquartile range 26) in the current study. The time intervals were also comparable between the LLR and OLR groups. The cross-sectional area of psoas muscles at the level of the inferior end plate of the third lumbar vertebrae was obtained from the CT scans. The areas (cm^2^) of bilateral psoas muscles were measured by a picture processing and analyzing software and further normalized by patients’ height squared (psoas muscle index (PMI) (cm^2^/m^2^) = total psoas muscle area (cm^2^)/height^2^ (m^2^)) [20, 35]. Both the preoperative and postoperative PMI were obtained, and PMI change was calculated by subtracting the preoperative PMI from the postoperative one. The change of PMI was further divided by preoperative PMI to yield the percentage change in PMI (percentage change in PMI (%) = (postoperative PMI — preoperative PMI)/preoperative PMI × 100%). All of the measurements and calculations were performed by the same research team who was blinded to the patient information.

### Metabolomic studies regarding significant muscle loss

For serum metabolomic analysis, we collected the patient’s serums at 1 month after the operation. The serum was processed and subjected to liquid chromatography-mass spectrometry (LC-MS) metabolomic analysis [36]. Since the current study aimed to investigate the degree of skeletal muscle loss after liver resection, the metabolomic study would preferentially focus on the major component of skeletal muscle, namely, amino acids. For statistical analysis, the patients were divided into three groups according to their decrease in PMI: ≤ 5%, 5–11.5%, and > 11.5%.

### Definition and statistical analysis

The preoperative preparation, operative procedures, and postoperative care had been described in our previous studies [6, 7]. Major liver resection was defined as liver resection of three or more segments according to the Brisbane 2000 terminology [37]. The difficulty levels were classified according to the 4-level IWATE criteria [38]. Surgical duration was defined as the time period elapsing from the induction of general anesthesia to endotracheal extubation The severity of postoperative complications was graded according to the Clavien-Dindo classification [39]. Significant muscle loss was defined as percentage change in PMI (%) more than “mean PMI change (%) - one standard deviation (%).” The sex-specific cutoff value for sarcopenia was defined as 6.36 cm^2^/m^2^ for men and 3.92 cm^2^/m^2^ for women in the current study [40]. The recurrence of HCC was determined by the emergence of image findings characteristic of HCC with or without elevated tumor markers during routine postoperative follow-up. Disease-free survival (DFS) was defined as the time interval from the date of surgery to the date of first documented disease recurrence. Tumor recurred within 2 years of liver resection was defined as early recurrence. The overall survival (OS) was defined as the time period spanning from the date of surgery to either the date of death or the date of last follow-up.

Continuous variables were expressed as mean ± standard deviation (SD) or median (interquartile range (IQR)) whenever appropriate and analyzed by Student’s *t*-test or Mann-Whitney *U*-test. Categorical variables were described by number and frequency (%) and analyzed by Pearson chi-square test. Kaplan-Meier method and long-rank test were adopted to analyze DFS and OS. Logistic regression analysis was employed to identify independent risk factors associated with significant muscle loss after liver resection. Cox proportional hazard model was adopted to explore prognostic factors predictive of early recurrence. Statistical significance was defined as *P*-value < 0.05. The statistical analysis was performed by IBM SPSS Statistics 22 (IBM Corporation, Software Group, Somers, NY, USA).

## Results

### Demographic characteristic of LLR and OLR for HCC

The patient demographics and clinical characteristics were similar between the OLR and LLR groups (all *P* > 0.05) (Table 1). The mean age of diagnosis was around 60 years, and the majority of patients were male. The mean BMI was 24 kg/m^2^, and about 36% of patients were categorized as sarcopenia before the operation. HBV infection was the most common etiology, and more than 40% of patients had liver cirrhosis. Laboratory parameters including hemoglobin, albumin, bilirubin, prothrombin time, neutrophil-to-lymphocyte ratio, and liver reserve were all comparable between the two groups.

**Table 1:** Patient characteristics

	LLR^a^ (*n* = 64 (100%))	OLR^b^ (*n* = 64 (100%))	*p*-value
Age (years) (mean ± SD^c^ (range))	60.2 ± 11.2	59.5 ± 11.8	0.729
Male gender, *n*(%)	49 (76.6)	52 (81.3)	0.516
DM^d^, *n*(%)	13 (20.3)	16 (25.0)	0.526
Hypertension, *n*(%)	17 (26.6)	22 (34.4)	0.337
ESRD^e^, *n*(%)	0 (0.0)	0 (0.0)	1.000
CCI^f^ score, median (IQR^g^)	5 (2.7)	6 (2.7)	0.162
ECOG^h^ ≧ 2	5 (7.8)	3 (4.7)	0.718
Smoking, *n*(%)	16 (25.0)	24 (37.5)	0.127
Alcohol, *n*(%)	13 (20.3)	18 (28.1)	0.302
BMI (kg/m^2^)^i^ (mean ± SD^c^)	24.9 ± 3.5	24.4 ± 3.3	0.441
Obesity, *n*(%)	15 (23.4)	15 (23.4)	1.000
Sarcopenia (pre-operative), *n*(%)	24 (37.5)	23 (35.9)	0.855
Previous abdominal surgery, *n*(%)	6 (9.4)	8 (12.5)	0.571
Preoperative treatment to HCC, *n*(%)	5 (7.8)	11 (17.2)	0.180
Child-Pugh class, *n*(%)			
Class A	64 (100)	64 (100)	1.000
BCLC stage, *n*(%)			1.000
Stage 0	11 (17.2)	12 (18.8)	
Stage A	51 (79.7)	51 (79.7)	
Stage B	2 (3.1)	1 (1.6)	
Preoperative ascites (*n*(%))	3 (4.7)	2 (3.1)	1.000
HBV infection, *n*(%)	37 (57.8)	43 (67.2)	0.273
HCV infection, *n*(%)	19 (29.7)	18 (28.1)	0.845
Liver cirrhosis, *n*(%)	27 (42.2)	36 (56.3)	0.157
Fatty liver, *n*(%)	31 (48.4)	27 (42.2)	0.478
Albumin (g/dL) (mean ± SD^c^)	4.20 ± 0.40	4.19 ± 0.41	0.533
Hemoglobin (gm/dL) (mean ± SD^c^)	13.7 ± 1.6	13.9 ± 1.7	0.503
NLR^j^, median (IQR^g^)	1.83 (0.98)	1.88 (0.85)	0.898
Platelet count (K/uL) (mean ± SD^c^)	174.7 ± 48.3	160.7 ± 53.8	0.121
T-bil (mg/dL), median (IQR^g^)	0.6 (0.5)	0.6 (0.5)	0.266
INR^k^ (mean ± SD^c^)	1.1 (0.1)	1.1 (0.11)	0.370
ICG-15^l^ > 10%, *n*(%)	20 (32.3)	21 (32.8)	0.947
α-Fetoprotein > 200 ng/mL, *n*(%)	15 (24.6)	11 (17.2)	0.308

^a^Laparoscopic liver resection, ^b^open liver resection, ^c^standard deviation, ^d^diabetes mellitus, ^e^end-stage renal disease, ^f^Charlson Comorbidity Index, ^g^iInterquartile range, ^h^Eastern Cooperative Oncology Group, ^i^body mass index, ^j^neutrophil-to-lymphocyte ratio, ^k^international normalized ratio, ^l^indocyanine green retention rate at 15 min

Supplementary Table S2 summarized the pathological features and surgical outcome of the current cohort. The tumor characteristics were essentially comparable between the LLR and OLR groups (all *P* > 0.05). The mean tumor size was around 3 cm, and more than 75% of tumors were encapsulated. Microscopic vascular invasion was identified in about 20%, and around 40% of tumors are categorized as Edmondson-Steiner grades 3 or 4. Stages 1 and 2 tumors comprised more than 90% of cases, and no patients enrolled had extrahepatic metastasis before the operation. Major liver resection accounted for 15% of procedures, and more than 50% of operations were classified as IWATE intermediate difficulty group.

The surgical outcome including surgical time, blood loss, and postoperative major complications, likewise, was comparable between the two groups. The length of postoperative hospital stay, on the other hand, was significantly shorter in the LLR group (9 days in the OLR and 7.5 days in the LLR, *P* < 0.001). While the preoperative albumin level was not different between the LLR and OLR, a significantly lower albumin level was found in the OLR group after the operation (3.46 g/dL in the OLR and 3.63 g/dL in the LLR, *P* < 0.001).

### Change of PMI in LLR and OLR groups

**Fig. 2:**
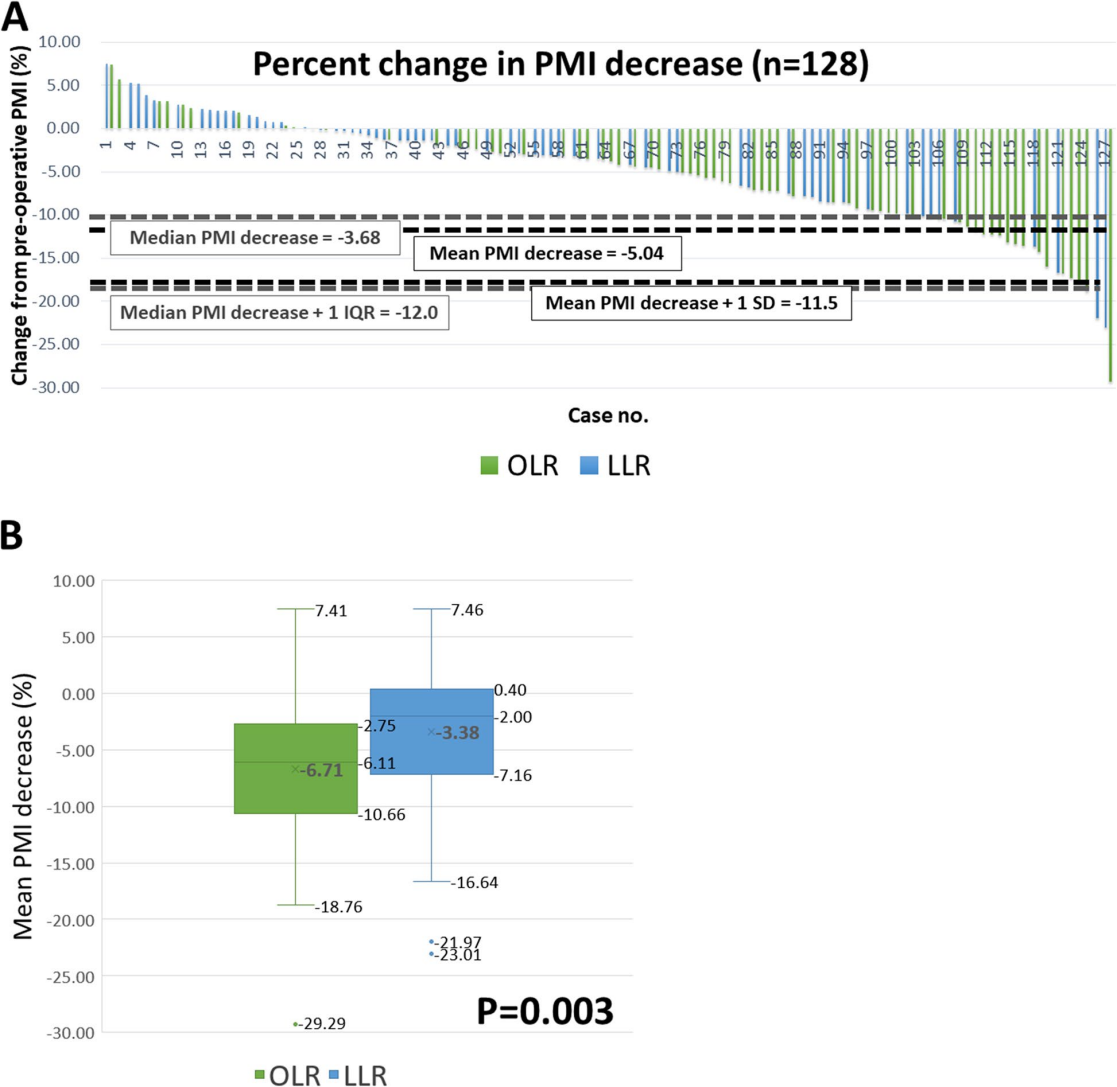
**A** Waterfall plot regarding the percent change in PMI after liver resection for HCC. **B** Mean PMI change (%) between LLR and OLR for HCC

The mean PMI change of the entire cohort was −5.0% with a standard deviation of 6.5% (Fig. 2A). Significant muscle loss was thus defined as more than “−5.0–6.5%,” which implied a reduction of more than 11.5% from the preoperative baseline PMI. Seventeen patients (13.3%) were found to have significant muscle loss after the operation. The PMI and muscle mass status both before and after surgery were summarized in Table 2. Although the perioperative PMI was similar between the two groups, OLR led to a more significant reduction in the PMI than the laparoscopic approach (mean change, −6.61% in the OLR group and −3.37% in the LLR group, *P* = 0.003) (Fig. 2B). The OLR group also had remarkably more significant muscle loss after surgery than the LLR group (21.9% vs. 4.7%, *P* = 0.008), which further impacted the prevalence of sarcopenia. Before liver resection, the prevalence of sarcopenia was comparable between the LLR and OLR groups. However, ten (15.4%) non-sarcopenic patients in the OLR group developed sarcopenia after surgery, in contrast to only 3 (4.7%) patients in the LLR group (*P* = 0.041).

**Table 2:** Change in psoas muscle after liver section for hepatocellular carcinoma

	LLR^a^ (*n* = 64 (100%))	OLR^b^ (*n* = 64 (100%))	*p*-value
PMI^c^ (pre-operative) (mean ± SD^d^)	6.50 ± 2.03	6.36 ± 1.55	0.663
PMI^c^ (post-operative) (mean ± SD^d^)	6.24 ± 1.87	5.93 ± 1.49	0.300
Change of PMI^c^ % (mean ± SD^d^)	−3.37 ± 5.92	−6.71 ± 6.50	0.003
Median (IQR^e^)	−1.98 ± 7.2	−5.92 ± 7.5	0.001
Significant muscle loss (> 11.5%), *n*(%)	3 (4.7%)	14 (21.9)	0.008
Sarcopenia (pre-operative), *n*(%)	24 (37.5)	23 (35.9)	0.855
Sarcopenia (post-operative), *n*(%)	26 (40.6)	31 (48.4)	0.374
De novo sarcopenia after operation, *n*(%)	3 (4.7)	10 (15.4)	0.041

^a^Laparoscopic liver resection, ^b^open liver resection, ^c^psoas muscle index (cm^2^/m^2^), ^d^standard deviation, ^e^interquartile range

### Predisposing factors of significant muscle loss

Univariate analysis was conducted to identify potential factors related to significant muscle loss following liver resection (Supplementary Tables S3 and S4). Laparoscopic approach was found to be associated with less significant muscle loss than the conventional open approach (*P* = 0.008). In addition, larger tumor size (*P* = 0.094), *AFP* > 200 ng/mL (*P* = 0.024), and tumor rupture (*P* = 0.017) were also correlated with significant muscle loss. Moreover, patients who experienced any grade of postoperative complications (*P* = 0.098) or major complications (*P* = 0.001) were more likely to have significant muscle loss after surgery. The length of postoperative hospital stay was longer among patients who developed significant muscle loss (11 vs. 8 days, *P* = 0.004). Other factors such as age, gender, comorbidity, performance status, body habitus, previous abdominal surgery, liver cirrhosis, liver biochemical function, tumor grade, tumor stage, extent of liver resection, and blood loss, on the other hand, were not related to the occurrence of significant muscle loss after surgery.

After logistic regression multivariate analysis, conventional open procedure, tumor rupture, *AFP* > 200 ng/mL, and occurrence of postoperative major complications were independent predisposing factors for the development of postoperative significant muscle loss (*P* = 0.01, 0.036, 0.004, and 0.003, respectively) (Table 3).

**Table 3:** Univariate and multivariate analysis of risks factors for significant muscle loss

	Univariate analysis		Multivariate analysis	
Variables	Odds ratio	*p*-value	Hazard ratio (95% *CI*^a^)	*p*-value
Operation approach (OLR vs LLR)	5.68	0.008	8.1 (1.6–39.0)	0.010
Tumor rupture (yes vs. no)	11.6	0.017	8.9 (1.15–66.6)	0.036
AFP^b^ > 200 ng/mL (yes vs. no)	3.27	0.026	7.4 (1.89–29.4)	0.004
Postoperative complication^c^ (any grade vs. none)	2.41	0.098	-	0.820
Grade ≧ III major complication^c^ (yes vs. no)	16.7	0.003	18.5 (2.73–125)	0.003
Postoperative length of stay (per day)	1.23	0.002	-	0.110
Tumor size (per cm)	1.34	0.082	-	0.901

^a^Confidence interval, ^b^α-fetoprotein, ^c^Clavien-Dindo classification

### Impact of significant muscle loss on the oncological outcome

**Fig. 3:**
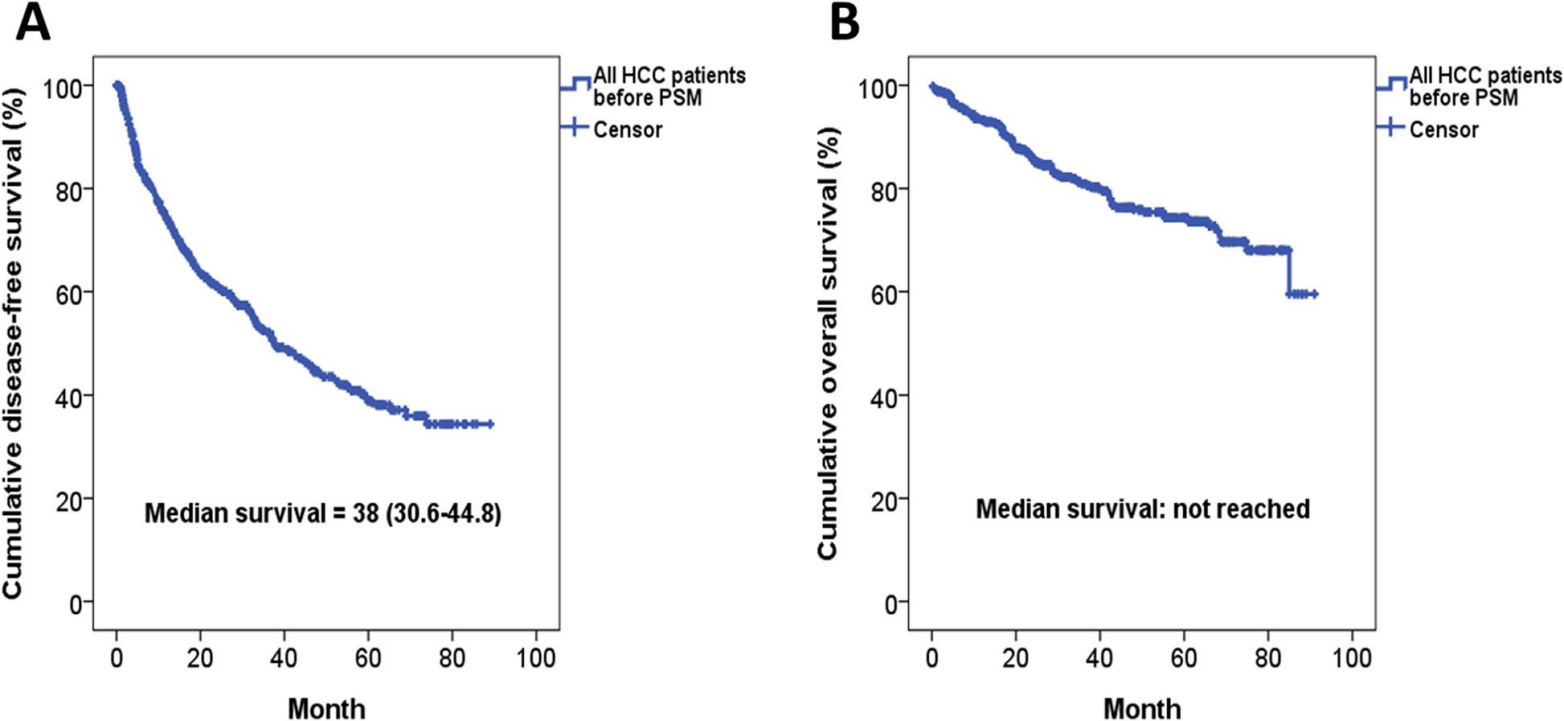
**A** Kaplan-Meier disease-free survival curve of the entire HCC cohort before propensity score-matched (PSM) analysis. **B** Kaplan-Meier overall survival curve of the entire HCC cohort before PSM analysis

**Fig. 4:**
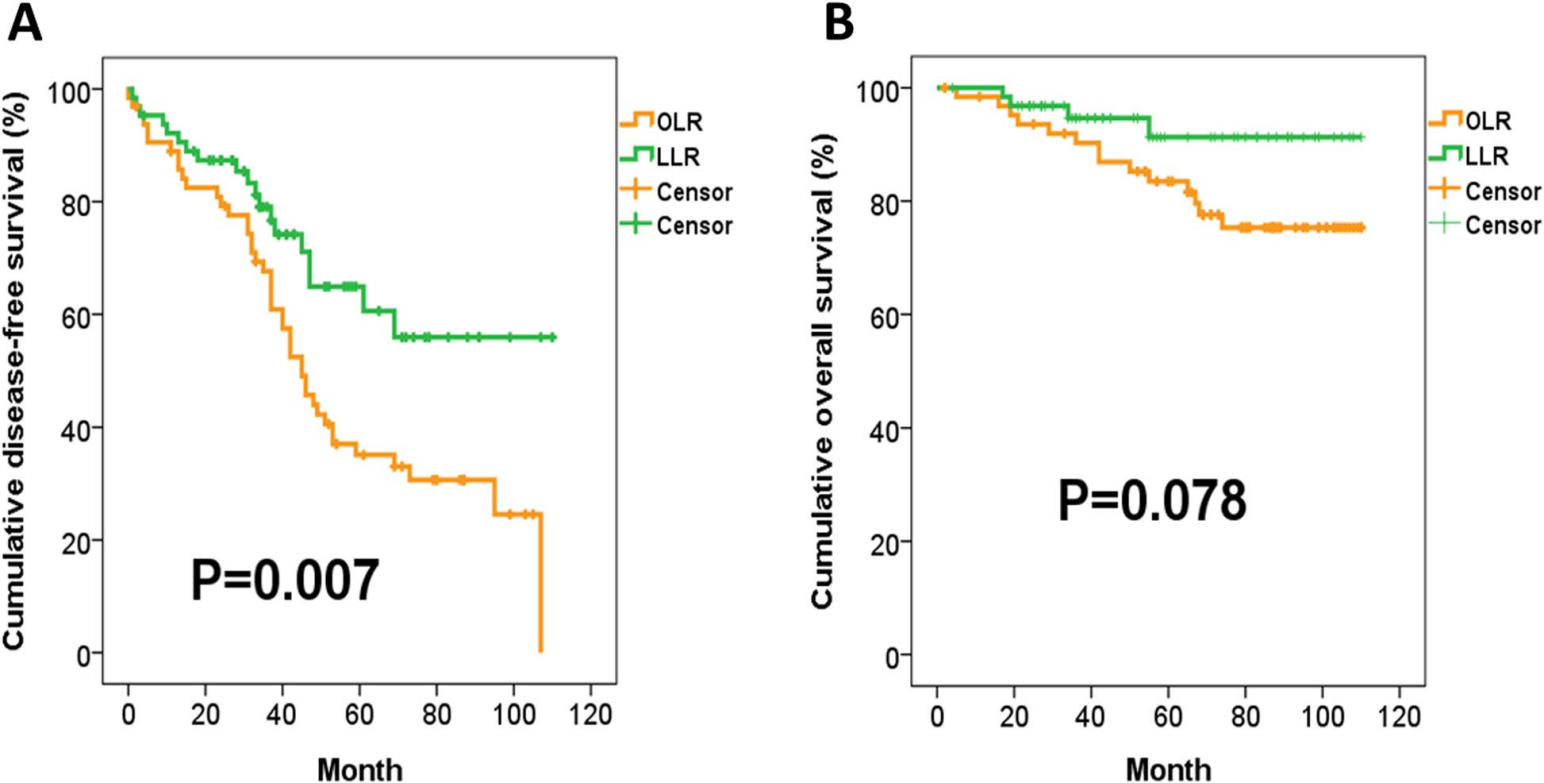
**A** Kaplan-Meier disease-free survival (DFS) curves of HCC patients receiving either laparoscopic liver resection (LLR) or open liver resection (OLR). The DFS of patients receiving LLR was significantly better than that of OLR. **B** Kaplan-Meier overall survival curves of HCC patients receiving either LLR or OLR. The OS of patients receiving LLR was marginally superior to that of OLR

**Fig. 5:**
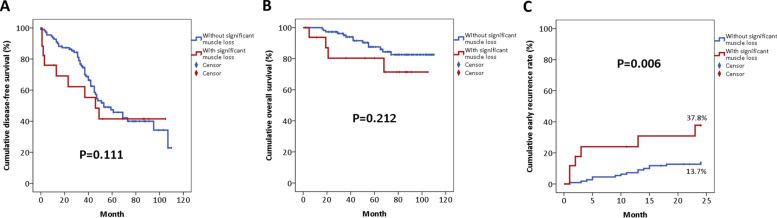
**A** Kaplan-Meier disease-free survival curves of HCC patients after surgery, stratified by significant muscle loss. **B** Kaplan-Meier overall survival curves of HCC patients after surgery, stratified by significant muscle loss. **C** Kaplan-Meier tumor recurrence curves of HCC patients after surgery, stratified by significant muscle loss. Early recurrence was defined as the occurrence of tumor relapse within 2 years of the index operation

The Kaplan-Meier DFS and OS curves of the entire HCC cohort before PSM were shown in Fig. 3A, B. The median DFS of the entire cohort was 38.0 months, and more than 50% of the patients were still alive at the end of this study. As for different surgical approach, the DFS of patients receiving LLR was significantly better than that of OLR (*P* = 0.007), and the OS of patients receiving LLR was marginally superior to that of OLR (*P* = 0.078) (Fig. 4A, B). Regarding the impact of muscle loss, Kaplan-Meier survival analysis demonstrated that the DFS and OS were comparable between patients with and without significant muscle loss (*P* = 0.111, *P* = 0.212, respectively) (Fig. 5A, B). However, there was a still trend toward favorable OS in patients without significant muscle loss. Moreover, the rate of early recurrence was significantly higher in patients suffering from significant muscle loss (37.8% vs. 13.7%, *P* = 0.006) (Fig. 3C).

The potential risk factors for early recurrence were further investigated and presented in Table 4. After analyzed by COX proportional hazards models with stepwise backward selection, type 2 diabetes mellitus (DM) (*HR* 3.05, *CI* 1.15–8.13, *P* = 0.025), ICG-15 > 10% (*HR* 4.48, *CI* 1.74–11.49, *P* = 0.002), significant muscle loss (*HR* 3.22, *CI* 1.17–8.84, *P* = 0.023), and AJCC cancer stage ≧ 3 (*HR* 7.41, *CI* 1.81–30.30, *P* = 0.005) were significantly related to early HCC recurrence.

**Table 4:** Univariate and multivariate analysis of risks factors for early recurrence

	Univariate analysis		Multivariate analysis		Stepwise backward selection	
Variables	Odds ratio (95% *CI*^a^)	*p*-value	Hazard ratio (95% *CI*^a^)	*p*-value	Hazard ratio (95%CI^a^)	*P* value
Age > 65 year	1.02 (0.41–2.53)	0.963	-	-	-	-
DM^b^	2.03 (0.78–4.80)	0.153	2.82 (7.51–1.06)	0.038	3.05 (1.15-8.13)	0.025
HBV^c^	1.21 (0.75–1.94))	0.431	-	-	-	-
HCV^d^	1.04 (0.62–1.61)	0.986	-	-	-	-
Liver cirrhosis	2.09 (0.84–5.17)	0.111	1.56 (0.58–4.16)	0.375	-	-
ICG-15^e^ > 10%	3.31 (1.39–7.87)	0.007	4.28 (1.62–11.30)	0.003	4.48 (1.74-11.49)	0.002
AFP^f^ > 200 ng/mL	2.28 (0.92–5.67)	0.074	1.83 (0.67–4.98)	0.231	-	-
Significant muscle loss	3.45 (1.33–8.91)	0.010	3.13 (1.08–9.02)	0.034	3.22 (1.17-8.84)	0.023
Pre-op sarcopenia	1.23 (0.49–3.04)	0.654	-	-	-	-
Laparoscopic procedure	0.59 (0.24–1.43)	0.246	-	-	-	-
Tumor rupture	3.58 (0.83–15.43)	0.086	1.16 (0.09–1.42)	0.905	-	-
Resection margin < 0.5 cm	1.42 (0.60–3.34)	0.421	-	-	-	-
Vessel invasion	1.82 (0.73–4.52)	0.194	-	-	-	-
AJCC^g^ cancer stage ≧ III	3.85 (1.13–13.11)	0.031	6.29 (0.71–55.21)	0.097	7.41 (1.81-30.30)	0.005
Major postoperative complication^h^	1.18 (0.15–8.85)	0.867	-	-	-	-

^a^Confidence interval, ^b^diabetes mellitus, ^c^hepatitis B virus, ^d^hepatitis C virus, ^e^indocyanine green retention rate at 15 min, ^f^α-fetoprotein, ^g^American Joint Committee on Cancer, ^h^Clavien-Dindo classification

### Metabolomic profiles of significant muscle loss

For metabolomic analysis, a total of 58 patients were finally enrolled. After LC-MS study, the concentrations of amino acids were compared between patients with the least and most PMI loss (≤ 5% vs. > 11.5%). As shown in Table 5, serum concentrations of ornithine and citrulline were significantly decreased in patients with PMI loss > 11.5%, while those of other amino acids were essentially comparable between patients with the least and most PMI loss.

**Table 5:** Serum amino acid profiles between patients with PMI^a^ decrease ≤ 5% and > 11.5%

Metabolites	PMI^a^ decrease ≤ 5%	PMI^a^ decrease > 11.5%	*p*-value
Ornithine	143.22 ± 42.92	103.48 ± 34.5	0.017
Citrulline	51.44 ± 28.22	37.62 ± 11.78	0.048
Threonine	130.18 ± 32.86	113.00 ± 29.41	0.173
Tyrosine	89.54 ± 23.64	78.62 ± 14.83	0.204
Glycine	216.47 ± 77.26	191.91 ± 54.58	0.386
Serine	128.94 ± 24.76	121.27 ± 22.76	0.418
Isoleucine	67.53 ± 22.59	74.04 ± 19.39	0.444
Tryptophan	36.80 ± 9.67	34.51 ± 10.79	0.554
Glutamine	446.48 ± 132.11	415.31 ± 146.42	0.554
Histidine	92.30 ± 14.23	89.29 ± 16.84	0.603
Asparagine	52.93 ± 13.73	50.08 ± 15.24	0.603

^a^Psoas muscle index

## Discussion

Surgical intervention can lead to loss of body skeletal muscle mass and strength due to many physiological perturbations including decreased physical activity, augmented stress response, increased metabolism, and interruption of essential nutritional support. Moreover, chronic liver disease or liver dysfunction would lead to altered protein turnover, malnutrition, deranged energy disposal, increased inflammation, and hormonal change, which in turn result in muscle depletion and occurrence of sarcopenia [41, 42]. Therefore, development of muscle loss following liver resection seemed inevitable and had been documented [26, 43, 44]. In addition to studies assessing muscle wasting following conventional open liver surgery, Himura et al. revealed that patients receiving laparoscopic liver resection would also experience muscle loss and deteriorated nutritional status, even in those without preoperative sarcopenia or severe postoperative complications [43]. There have also been other studies investigating the impact of surgery on skeletal muscle mass and subsequent survival in patients with different types of cancers [44] (Supplementary Table S5). However, the impact of different surgical approach on muscle change after liver resection remained undetermined. The present study is by far the first one in the English literature which compared the extent of muscle loss between conventional open and laparoscopic liver resection for HCC. According to the current study, the PMI decreased more significantly in patients receiving OLR than LLR. Given inherent merits of minimally invasive procedure, laparoscopic approach was expected to produce less stress response and better postoperative physical activity, thus subsequently reducing surgery-related muscle loss.

In the current study, we have demonstrated that conventional open procedure was an independent risk factor for the development of significant muscle loss. Laparoscopic liver resection for HCC, on the other hand, was associated with less significant muscle loss. The advantage of laparoscopic approach in preventing muscle wasting has also been reported in patients with gastric cancer. Compared to open gastrectomy, laparoscopic gastrectomy was associated with enhanced recovery of muscle mass at 6 months after surgery [45]. In addition, there were other studies revealing the protective effect of laparoscopic procedure on surgery-related muscle loss [26, 46]. Furthermore, the current study found that serum albumin level on the 7th postoperative day was significantly lower in patients receiving open surgery. Our finding paralleled a previous report that laparoscopic procedure may reduce the development of hypoalbuminemia and ascites by protecting the collateral vein circulation of the abdominal wall [47]. Since albumin is known to indicate the global nutritional status, and hypoalbuminemia, similar to sarcopenia, has been found to be predictive of early postoperative outcome after liver resection and its drop is also a marker of stress response, laparoscopic liver resection thus can maintain a better nutritional profile and produce less surgery-related stress response [48, 49]. As a result, the benefits of laparoscopic surgery for HCC could include not only smaller wounds, earlier recovery, less pain, and shorter hospital stay but also more favorable body composition!

In addition to surgical approach, the current study revealed that tumor rupture, larger tumor, *AFP* > 200 ng/mL, and major complications also predisposed to the development of significant muscle loss. Similar to pancreatic cancer, in which a higher preoperative CA 19-9 was associated with surgical-related muscle loss [22], we have discovered that a more advanced HCC in terms of AFP or tumor size could also result in more pronounced muscle loss after surgery. Since disease severity is intrinsic to tumors and can hardly be altered, we should thus try to optimize the modifiable variables, i.e., adoption of laparoscopic approach and avoidance of major complications, to minimize skeletal muscle loss after surgery [24, 26, 46].

From the current study, significant muscle loss could impact not only the perioperative recovery but also the oncological outcome. Early recurrence, according to our analysis, was remarkably more likely to occur in patients suffering from significant muscle loss. Since it has been demonstrated that early recurrence was closely related to OS in HCC, clinicians therefore should extreme their efforts to prevent the development of early recurrence in order to prolong OS [50, 51]. Laparoscopic liver resection for HCC, as a result, could be a more favorable surgical approach to reduce the risk of early recurrence by avoiding significant skeletal muscle loss. The mechanism underlying early tumor recurrence, on the other hand, remained undetermined. Whether occult tumor metastasis or impaired immunity may play a role deserves further investigation.

In addition to early tumor recurrence, the current study also discovered that patients having significant muscle loss had considerably lower serum concentrations of ornithine and citrulline after surgery. Both ornithine and citrulline are intermediate metabolites of the urea cycle. This cycle converts highly toxic ammonia to urea for excretion and primarily takes place in the liver [52, 53]. The reduction of ornithine and citrulline after surgery indicated reduced urea cycle, which might represent declined liver function [54]. Furthermore, studies have shown that citrulline was capable of protecting against skeletal muscle wasting by activating the machinery of muscle protein synthesis [55, 56]. Supplementation of citrulline in aged malnourished rats could promote skeletal muscle protein synthesis during periods of malnourishment or low protein intake [57]. The reduced uremic metabolites in human blood were also prominent feature of sarcopenia [58]. As a result, it is speculated that the significant muscle loss after surgery may partly be attributed to diminished citrulline. Whether this citrulline reduction was related to impaired liver ureotelic function remains uncertain and mandates further investigation.

Despite remarking findings, the present study still had several limitations. First, the definition of significant muscle loss was not well established. A 10% decrease in PMI was regarded significant in several cancers including esophageal cancer, pancreatic cancer, and colorectal cancer [22, 23, 25, 59]. In patients with end-stage liver disease receiving living donor liver transplantation, a decrease in *PMI* ≥ 11.7% was classified as high muscle loss [60]. The current study, in which significant muscle loss was defined as percentage change in PMI (%) more than “mean PMI change (%) - one standard deviation (%) = 11.5%,” was similar to previous reports. However, further study is still warranted to precisely define the cutoff for significant muscle loss. Secondly, although PSM has been adopted, selection bias was still inevitable in a retrospective study. Patients who lacked critical clinical data were also unavoidably excluded. The relatively small sample size similarly rendered our findings less convincing. Moreover, the present study examined muscle quantity in terms of psoas muscle area. The quality of skeletal muscle, on the other hand, was not investigated. Future larger scale prospective randomized studies investigating both muscle mass, strength, and quality thus are mandatory to validate our findings. Furthermore, the current study only adopted the IWATE difficulty score to assess the complexity of liver tumors. Other promising and validated scoring systems, for example, the IMM score [61, 62], were not considered. We believe these novel systems should be incorporated in the future studies to precisely describe the difficulty of liver resections. Last but not the least, the clinical significance of decreased citrulline and ornithine in patients with significant muscle loss remained undetermined. The efficacy of citrulline/ornithine supplementation in preventing or restoring muscle loss after surgery also deserves further investigation. Future trials are therefore required to answer these questions.

## Conclusion

In conclusion, our study demonstrated that laparoscopic liver resection was associated with less significant muscle loss than the conventional open approach for HCC patients undergoing liver resection. Significant muscle loss, on the other hand, was a predictive factor for early tumor recurrence after surgery. In addition, it was associated with deranged metabolism of certain amino acids. As a result, laparoscopic HCC resection should lead to a more favorable outcome and must be considered for patients eligible for LLR. Future studies are warranted to validate our findings.

## Supplementary Information

**Additional file 1: Supplementary Table S1.** The clinicopathological data of the entire cohort.

**Additional file 2: Supplementary Table S2.** Surgical and pathological characteristics.

**Additional file 3: Supplementary Table S3.** Clinical characteristics of patients with significant muscle loss after liver resection.

**Additional file 4: Supplementary Table S4.** Surgical and pathological characteristics of patients with significant muscle loss after liver resection.

**Additional file 5: Supplementary Table S5.** Summary of studies regarding muscle loss after surgery.

## Data Availability

All data generated or analyzed during the study are included in this published article. Raw data may be requested from the authors with the permission of the institution.

## Abbreviations

AFP: α-Fetoprotein
AJCC: American Joint Committee on Cancer
BMI: Body mass index
CCI: Charlson Comorbidity Index
CGMH: Chang Gung Memorial Hospital
CI: Confidence interval
DFS: Disease-free survival
DM: Diabetes mellitus
DS: Difficulty score
ECOG: Eastern Cooperative Oncology Group
ESRD: End-stage renal disease
Hb: Hemoglobin
HBV: Hepatitis B virus
HCV: Hepatitis C virus
HCC: Hepatocellular carcinoma
ICG-15: Indocyanine green retention rate at 15 min
IQR: Interquartile range
IRB: Institutional review board
LLR: Laparoscopic liver resection
LOS: Length of stay
NLR: Neutrophil-to-lymphocyte ratio
OLR: Open liver resection
OS: Overall survival
PMI: Psoas muscle index
POD: Postoperative day
PSM: Propensity score matching
SD: Standard deviation
