# Supplementary material for: Laparoscopic liver resection is associated with less significant muscle loss than the conventional open approach
Source: World J Surg Oncol. 2022 Dec 4;20:385. doi: 10.1186/s12957-022-02854-1 (PMC9721003; doi:10.1186/s12957-022-02854-1)
Supplement: Supplementary file 1 — Additional file 1: Supplementary Table S1. The clinicopathological data of the entire cohort. [file 12957_2022_2854_MOESM1_ESM.docx]

**Supplementary Table S1. The clinicopathological data of the entire cohort**

| Variables | LLR (N=90(100%)) | OLR (N=506(100%)) | *P* value |
| --- | --- | --- | --- |
| Age (year) (Mean±SD) | 60.4±12.1 | 61.7±11.4 | 0.167 |
| Male gender, n(%) | 66 (73.3) | 394 (77.8) | 0.533 |
| Previous abdominal surgery, n(%) | 10 (11.1) | 163 (30.9) | <0.001 |
| DM^c^, n(%) | 20 (22.1) | 142 (28.0) | 0.273 |
| Hypertension, n(%) | 28 (31.8) | 207 (41.1) | 0.055 |
| ESRD^d^, n(%) | 0 (0.0) | 4 (0.7) | 0.788 |
| HBV infection, n(%) | 51 (56.6) | 304 (60.1) | 0.891 |
| HCV infection, n(%) | 32 (35.5) | 131 (25.8) | 0.172 |
| Cirrhosis, n(%) | 39 (43.3) | 245 (48.4) | 0.521 |
| Hemoglobin (g/dL) (Mean±SD) | 13.6±1.9 | 13.5±1.9 | 0.872 |
| Albumin (g/dL) (Mean±SD) | 4.19±0.42 | 4.16±0.41 | 0.720 |
| ICG-15 > 10%, n(%) | 37 (41.2) | 213 (42.1) | 0.882 |
| α-fetoprotein > 200 ng/mL, n(%) | 24 (26.6) | 127 (25.1) | 0.612 |
| Child-Pugh classification, n(%) |  |  | 1.000 |
| A | 90 (100.0) | 503 (99.5) |  |
| B | 0 (0.0) | 3 (0.5) |  |
| Tumor size (cm) (Mean±SD) | 3.17±1.46 | 5.14±3.82 | <0.001 |
| Tumor size >3cm, n(%) | 44 (48.8) | 329 (65.0) | 0.006 |
| Tumor size >5cm, n(%)) | 7 (7.7) | 160 (31.6) | <0.001 |
| Major resection^e^, n(%) | 14 (15.5) | 171 (33.7) | <0.001 |
| Difficulty score group, n(%) |  |  | <0.001 |
| Low (score 0-3) | 20 (22.2) | 29 (5.7) |  |
| Intermediate (score 4-6) | 48 (53.3) | 126 (24.9) |  |
| Advanced (score 7-9) | 17 (18.8) | 206 (40.7) |  |
| Expert (score 10-13) | 6 (6.6) | 145 (28.6) |  |
| AJCC T Stage, n(%) |  |  | 0.003 |
| T1 | 56 (62.2) | 240 (47.4) |  |
| T2 | 29 (32.2) | 154 (30.4) |  |
| T3a | 0 (0.0) | 31 (6.1) |  |
| T3b | 3 (3.3) | 32 (5.7) |  |
| T4 | 2 (2.2) | 49 (9.6) |  |

^a^ laparoscopic liver resection ^b^ open liver resection ^c^ diabetes mellitus ^d^ end-stage renal disease ^e^ resection of more than 3 segments
