# Supplementary material for: Laparoscopic liver resection is associated with less significant muscle loss than the conventional open approach
Source: World J Surg Oncol. 2022 Dec 4;20:385. doi: 10.1186/s12957-022-02854-1 (PMC9721003; doi:10.1186/s12957-022-02854-1)
Supplement: Supplementary file 2 — Additional file 2: Supplementary Table S2. Surgical and pathological characteristics. [file 12957_2022_2854_MOESM2_ESM.docx]

**Supplementary Table S2. Surgical and pathological characteristics**

|  | LLR^a^  (n = 64 (100%)) | OLR^b^  (n = 64 (100%)) | *P* value |
| --- | --- | --- | --- |
| Pathological variables | | | |
| Tumor size (cm) (Mean ± SD^c^)) | 3.02 ± 1.16 | 2.98 ± 1.51 | 0.486 |
| Tumor size >5cm, n(%) | 4 (6.3) | 6 (9.4) | 0.744 |
| Rupture, n(%) | 2 (3.1) | 3 (4.7) | 1.000 |
| Necrosis, n(%) | 24 (37.5) | 26 (40.6) | 0.717 |
| Tumor with capsule, n(%) | 53 (82.8) | 49 (76.6) | 0.416 |
| Capsule invasion, n(%) | 43 (67.2) | 39 (60.9) | 0.461 |
| Vessel invasion, n(%) | 16 (25.0) | 12 (18.8) | 0.392 |
| Tumor grade ≧ III, n(%) | 24 (37.5) | 26 (40.6) | 0.717 |
| Section margin <0.5cm, n(%) | 25 (39.1) | 26 (40.6) | 0.857 |
| Major resection, n(%) | 10 (15.6) | 9 (14.1) | 0.804 |
| Difficulty group^d^, n(%) |  |  | 0.152 |
| Low | 7 (10.9) | 3 (4.7) |  |
| Intermediate | 35 (54.7) | 34 (53.1) |  |
| High | 15 (23.4) | 24 (37.5) |  |
| Expert | 7 (10.9) | 3 (4.7) |  |
| AJCC Stage, n(%) |  |  | 0.126 |
| Stage I | 41 (64.1) | 47 (73.4) |  |
| Stage II | 21 (32.8) | 12 (18.8) |  |
| Stage III | 2 (3.1) | 5 (7.8) |  |
| Stage IV | 0 (0.0) | 0 (0.0) |  |
| Surgical outcome | | | |
| Surgical time (min) (Mean ± SD) | 282.9 ± 99.9 | 265.0 ± 90.8 | 0.290 |
| Blood loss >500 ml, n(%) | 18 (28.1) | 18 (28.1) | 1.000 |
| Post-operative length of stay (LOS)(day), median (IQR^e^) | 7.5 (2.0) | 9 (3.0) | <0.001^￡^ |
| Post-operative complication^f^ (any grade) , n(%) | 15 (23.4) | 17 (26.6) | 0.683 |
| Ascites, n(%) | 3 (4.7) | 7 (10.9) |  |
| Pleural effusion, n(%) | 1 (1.6) | 3 (4.7) |  |
| Bile leakage, n(%) | 2 (3.1) | 2 (3.1) |  |
| Grade≧III major complication^f^, n(%) | 1 (1.6) | 5 (7.8) | 0.208 |
| Post-operative albumin supplement, n(%) | 11 (18.3) | 17 (30.4) | 0.130 |
| Albumin (g/dL) on POD7^g^, median (IQR^e^) | 3.63 (0.41) | 3.46 (0.54) | <0.001^￡^ |
| NLR^h^ on POD7, median (IQR^e^) | 3.01 (2.06) | 3.3 (1.96) | 0.151^￡^ |

^a^ laparoscopic liver resection ^b^ open liver resection ^c^ standard deviation ^d^ IWATE criteria ^e^ interquartile range ^f^ Clavien-dindo classification ^g^ Post-operative day ^h^ Neutrophil-to-Lymphocyte ratio
