# Supplementary material for: Laparoscopic liver resection is associated with less significant muscle loss than the conventional open approach
Source: World J Surg Oncol. 2022 Dec 4;20:385. doi: 10.1186/s12957-022-02854-1 (PMC9721003; doi:10.1186/s12957-022-02854-1)
Supplement: Supplementary file 3 — Additional file 3: Supplementary Table S3. Clinical characteristics of patients with significant muscle loss after liver resection. [file 12957_2022_2854_MOESM3_ESM.docx]

**Supplementary Table S3. Clinical characteristics of patients with significant muscle loss after liver resection**

|  | With significant PMI decrease (n (%)) | *P* value |
| --- | --- | --- |
| Age (≧ 65 vs. < 65 year-old) | 7 (15.9) vs. 10 (11.9) | 0.526 |
| Gender (male vs female) | 13 (12.9) vs. 4 (14.8) | 1.000 |
| DM^b^ (yes vs. no) | 3 (10.3) vs. 14 (14.1) | 0.761 |
| Hypertension (yes vs. no) | 5 (12.8) vs. 12 (13.5) | 0.183 |
| CCI^c^ score (Mean ± SD^d^ ) | 5.17 ± 1.55 vs. 5.27 ± 1.70 | 0.815 |
| ECOG^e^ (≧ 2 vs < 2) | 1 (12.5) vs. 16 (13.3) | 1.000 |
| Smoking (yes vs. no) | 5 (12.5) vs. 12 (13.6) | 0.861 |
| Alcohol (yes vs. no) | 6 (19.4) vs. 11 (11.3) | 0.252 |
| BMI^f^ (Mean ± SD) | 24.1 ± 3.7 vs. 24.7 ± 3.4 | 0.476 |
| Obesity (BMI ≧ 27 vs < 27) | 4 (13.3) vs.13 (13.3) | 1.000 |
| Sarcopenia (pre-operative) (yes vs. no) | 4 (8.5) vs. 13 (16.0) | 0.286 |
| Previous abdominal surgery history (yes vs. no) | 1 (7.1) vs. 16 (14.0) | 0.692 |
| Pre-operative treatment to HCC^g^ (any vs. none) | 2 (12.5) vs. 15 (13.4) | 0.922 |
| HBV infection (positive vs. negative) | 10 (12.5) vs. 7 (14.6) | 0.737 |
| HCV infection (positive vs. negative) | 6 (16.2) vs. 11 (12.1) | 0.533 |
| Liver cirrhosis (yes vs. no) | 9 (14.3) vs. 8 (12.3) | 0.742 |
| Fatty liver (yes vs. no) | 7 (12.1) vs. 10 (14.3) | 0.713 |
| Albumin (g/dL) (Mean ± SD) | 4.1 ± 0.4 vs. 4.2 ± 0.4 | 0.345 |
| Hemoglobin (gm/dL) (Mean ± SD) | 13.3 ± 1.5 vs. 13.9 ± 1.7 | 0.175 |
| NLR^h^, median (IQR^i^) | 1.58 (0.9) vs. 1.88 (0.9) | 0.773^￡^ |
| Platelet count (K/uL) (Mean ± SD) | 155 ± 64.1 vs. 169.6 ± 48.8 | 0.301 |
| T-bil (mg/dL), median (IQR^i^) | 0.7 (0.6) vs. 0.6 (0.5) | 0.252^￡^ |
| INR, median (IQR^i^) | 1.1 (0.10) vs 1.1 (0.15) | 0.145^￡^ |
| ICG-15 (≧ 10% vs. < 10%) | 8 (19.5) vs. 9 (10.6) | 0.170 |
| α-fetoprotein (≧ 200 vs. < 200 ng/mL) | 7 (26.9) vs. 10 (10.0) | 0.024 |

^a^ psoas muscle index ^b^ diabetes mellitus ^c^ Charlson comorbidity index ^d^ standard deviation ^e^ Eastern Cooperative Oncology Group ^f^ body mass index ^g^ Hepatocellular carcinoma ^h^ eutrophil-to-Lymphocyte ratio ^i^ interquartile range
