# Supplementary material for: Laparoscopic liver resection is associated with less significant muscle loss than the conventional open approach
Source: World J Surg Oncol. 2022 Dec 4;20:385. doi: 10.1186/s12957-022-02854-1 (PMC9721003; doi:10.1186/s12957-022-02854-1)
Supplement: Supplementary file 4 — Additional file 4: Supplementary Table S4. Surgical and pathological characteristics of patients with significant muscle loss after liver resection. [file 12957_2022_2854_MOESM4_ESM.docx]

**Supplementary Table S4. Surgical and pathological characteristics of patients with significant muscle loss after liver resection**

|  | With significant PMI decrease (n (%)) | *P* value |
| --- | --- | --- |
| Pathological variables |  |  |
| Tumor size (cm), median (IQR^b^) | 3.4 (2.4) vs. 2.8 (1.6) | 0.094^￡^ |
| Tumor size (≧ 5 vs. < 5 cm) | 3 (30) vs. 14 (11.9) | 0.129 |
| Tumor number (single vs. multiple) | 16 (13.8) vs. 1 (9.1) | 0.841 |
| Rupture (yes vs. no) | 3 (60.0) vs. 14 (11.4) | 0.017 |
| Necrosis (yes vs. no) | 9 (18.0) vs. 8 (10.3) | 0.208 |
| Tumor with capsule (yes vs. no) | 17 (15.0) vs. 0 (0.0) | 0.218 |
| Capsule invasion (yes vs. no) | 12 (14.6) vs. 5 (10.9) | 0.547 |
| Vessel invasion (yes vs. no) | 5 (17.9) vs. 12 (12.0) | 0.420 |
| Tumor grade (≧ III vs I&II) | 6 (12.0) vs. 11 (14.1) | 0.732 |
| Section margin (< 0.5 vs. ≧ 0.5cm) | 7 (13.7) vs. 10 (13.0) | 0.904 |
| Major hepatic resection (major vs. minor) | 3 (15.8) vs. 14 (12.8) | 0.727 |
| Approach procedure (laparoscopic vs. open) | 3 (4.7) vs. 14 (21.9) | 0.008 |
| AJCC^c^ Stage (I vs. II&III) | 11 (12.5) vs. 6 (15.0) | 0.699 |
| AJCC Stage (I&II vs. III) | 15 (12.4) vs. 2 (28.6) | 0.233 |
| Surgical factors |  |  |
| Surgical time (≧ 200 vs. < 200 min) | 13 (13.0) vs.4 (14.3) | 0.859 |
| Blood loss (≧ 500 vs. < 500 ml) | 4 (11.1) vs.13 (14.3) | 0.777 |
| Post-operative length of stay (LOS)(day), median (IQR^b^) | 11 (6.0) vs. 8 (3.0) | 0.004^￡^ |
| Post-operative complication^d^ (any vs. none) | 7 (21.9) vs. 10 (10.4) | 0.098 |
| Ascites, n(%) | 2 |  |
| Pleural effusion, n(%) | 1 |  |
| Bile leakage, n(%) | 2 |  |
| Grade ≧ III major complication^d^ (yes vs. no) | 4 (66.7) vs. 13 (10.7) | 0.001 |
| Post-operative albumin supplement, n(%) | 6 (21.4) vs. 9 (10.2) | 0.124 |
| Albumin (g/dL) on POD7^e^, median (IQR^b^) | 3.51 (0.89) vs. 3.55 (0.48) | 0.347^￡^ |
| NLR^f^ on POD7, median (IQR^b^) | 3.19 (2.29) vs. 3.14 (1.92) | 0.798^￡^ |

^a^ psoas muscle index ^b^ interquartile range ^c^American Joint Committee on Cancer ^d^ Clavien-dindo classification ^e^ Post-operative day ^f^ Neutrophil-to-Lymphocyte ratio
