# Supplementary material for: Laparoscopic liver resection is associated with less significant muscle loss than the conventional open approach
Source: World J Surg Oncol. 2022 Dec 4;20:385. doi: 10.1186/s12957-022-02854-1 (PMC9721003; doi:10.1186/s12957-022-02854-1)
Supplement: Supplementary file 5 — Additional file 5: Supplementary Table S5. Summary of studies regarding muscle loss after surgery. [file 12957_2022_2854_MOESM5_ESM.docx]

**Supplementary Table S5. Summary of studies regarding muscle loss after surgery**

| Authors | Year | Patients number / Surgical disease | Mean muscle loss | Cutoff of significant muscle loss | Primary outcome | Predisposing factors |
| --- | --- | --- | --- | --- | --- | --- |
| Argillander *et al.^44^* | 2021 | 233 / Non-metastatic colorectal cancer | −8.1% (− 28.2% to −5.8%) | > 5.7% | Higher 3-year mortality rate (27% vs 14%, *P* = 0.05) and reduced OS*^c^* (HR 2.8, *P* =0.002) in patients with muscle wasting | N.A. *^a^* |
| Choi *et al.^22^* | 2018 | 180 / Pancreatic cancer | N.A.*^a^* | >10%/60 days | Poorer OS*^c^* (*P* = 0.029) | For muscle loss: CA 19-9, LOS*^f^*, length of ICU*^g^* stay  For OS*^c^*: Preoperative sarcopenia, adjuvant chemotherapy, lymph node metastasis, poor differentiation |
| Kawakita *et al.^24^* | 2020 | 113 / Esophageal cancer | -12.2% (3.0–19.7%) | >20% | Poorer overall (*P*<0.0001) and recurrence-free (*P* = 0.0097) survivals | For muscle loss: operation time, anastomotic leakage, LOS*^f^*  For OS*^c^*: male gender, initial low BMI*^h^*, initial CAR*^i^* |
| Maeda *et al.^46^* | 2020 | 72 / Esophageal cancer | -4.4% (-10 to -0.8%) on POD3*^b^* | >4.4% | Worse 3-year OS*^c^* (*P* = 0.0128) | For muscle loss: open procedure (*vs.* thoracoscopic), blood loss  For OS *^c^*: pathologic stage, preoperative therapy |
| Park *et al.^25^* | 2017 | 58 / Esophageal cancer | −10.17% | >10% | Poorer OS*^c^* (*P* = 0.048)  3-year OS*^c^* 18.9% *vs.*58.2% (*P* = 0.049) | For OS*^c^*: muscle loss, stage III |
| van Wijk *et al.^26^* | 2021 | 128 / Liver resection for colorectal cancer liver metastasis | −7.1% ± 5.7% | >7.1% | Significantly lower OS*^c^* in patients with muscle quality loss (*P* = 0.012) | For muscle loss in quantity: resection *vs.* resection+RFA*^j^*, DM*^k^*, COPD*^l^*  For muscle loss in quality: open procedure, operation time, age |
| Yamazaki *et al.^57^* | 2020 | 167 / Laparoscopic gastrectomy for gastric cancer | N.A.*^a^* | >10% | Median muscle loss: 5.9% in the LDG*^d^* and 15.6% in the LTG*^e^* (1 postoperative year) | For muscle loss: LTG *^e^* and postoperative respiratory complications |

*^a^* not applicable *^b^* postoperative day 3 *^c^* overall survival *^d^* laparoscopic distal gastrectomy *^e^* laparoscopic total gastrectomy *^f^* length of stay *^g^* intensive care unit *^h^* body mass index *^i^* C-reactive protein-to-albumin ratio *^j^* radiofrequency ablation *^k^* diabetes mellitus *^l^* chronic obstructive pulmonary disease
